# One nose, one brain: contribution of the main and accessory olfactory system to chemosensation

**DOI:** 10.3389/fnana.2012.00046

**Published:** 2012-11-09

**Authors:** Carla Mucignat-Caretta, Marco Redaelli, Antonio Caretta

**Affiliations:** ^1^Department of Molecular Medicine, University of PadovaPadova, Italy; ^2^Department of Pharmaceutical Sciences, University of ParmaParma, Italy

**Keywords:** vomeronasal organ, nose, olfaction, pheromones

## Abstract

The accessory olfactory system is present in most tetrapods. It is involved in the perception of chemical stimuli, being implicated also in the detection of pheromones. However, it is sensitive also to some common odorant molecules, which have no clear implication in intraspecific chemical communication. The accessory olfactory system may complement the main olfactory system and may contribute different perceptual features to the construction of a unitary representation, which merges the different chemosensory qualities. Crosstalk between the main and accessory olfactory systems occurs at different levels of central processing, in brain areas where the inputs from the two systems converge. Interestingly, centrifugal projections from more caudal brain areas are deeply involved in modulating both main and accessory sensory processing. A high degree of interaction between the two systems may be conceived and partial overlapping appears to occur in many functions. Therefore, the central chemosensory projections merge inputs from different organs to obtain a complex chemosensory picture.

## Introduction

**Figure 1:**
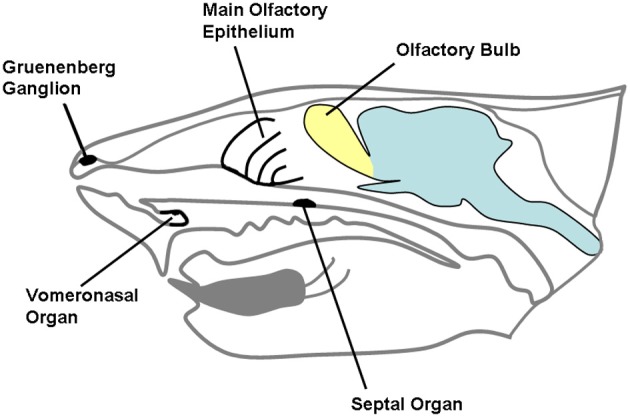
**A schematic representation of rodent head in the sagittal plane.** The different chemosensory systems of the nasal cavity are indicated.

Chemical substances originating from the environment stimulate a variety of receptors hosted in the nasobuccal cavity. While taste and trigeminal sensitivity of the mouth are mainly related to evaluation of food, chemical senses of the nose are devoted to evaluation of external stimuli, related to different environmental aspects, including the detection of dangerous substances, food retrieval, and social interactions (Ma, 2007). The chemical senses of mammalian nose include the main olfactory epithelium and the vomeronasal organ (VNO), that are complemented in their functions by the trigeminal afferents and the receptors located in the organ of Masera and in the ganglion of Gruenenberg (Figure 1); the function of the last three supports olfaction in detection of molecules (Breer et al., 2006). Complex interactions are now emerging between the main and accessory olfactory systems, both at anatomical and at functional level.

**Figure 2:**
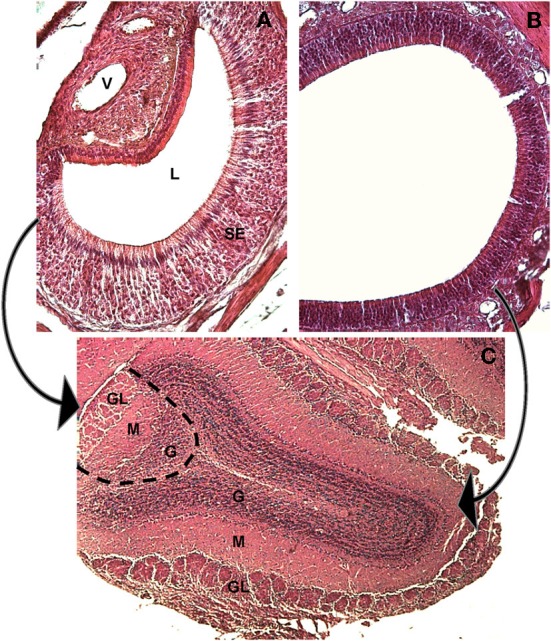
**The main and accessory olfactory epithelia of the mouse, and their first central targets, the main and accessory olfactory bulbs.** Hematoxylin-eosin staining, mouse at 30 days of age. **(A)** A coronal section of the VNO, medial to the right, lateral to the left, dorsal to the top. The sensory epithelium (SE), the lumen (L) and the vessel (V) are indicated. **(B)** A coronal section of the main olfactory epithelium, dorsal to the upper right. **(C)** A horizontal section through the main and accessory olfactory bulb. The dotted line represents the border between the MOB (to the right) and the AOB (to the left). The arrows indicate the projections from the MOE to the main olfactory bulb (right) and from the VNO to the AOB (left). In both bulbs the main structures are indicated: (GL) glomerular layer, (M) mitral cell layer, (G) granular layer.

The main olfactory system originates from the olfactory mucosa and projects to the main olfactory bulb (MOB), while the VNO, described by Jacobson in 1813 (Trotier and Doving, 1998) projects to the accessory olfactory bulb (AOB, see Figure 2).

The VNO appears as a derived character in tetrapods (Eisthen, 1992): it is present already in aquatic species from the Devonian period, before transition to land (Swaney and Keverne, 2009). Later, it has been lost in some species, including crocodilians, cetaceans, some bats, and primates. The accessory olfactory system may differ in morphology even in closely related species, most probably in relation to functional specialization (for a review, see Salazar and Quinteiro, 2009).

### Hypothesis on the function of the accessory olfactory system

Since its first description by Jacobson [translated in Trotier and Doving (1998)], the VNO has been described as a sensory organ, possibly in support of the sense of smell, but its precise role in chemosensation is still poorly understood. The first functional investigation of the accessory olfactory system pointed to a general chemosensation role. One old anatomical study suggested that the VNO, being filled with mucus, should be sensitive to water-borne molecules (Broman, 1920). Electrophysiological recordings preceded any other functional testing, and suggested that the VNO was activated by mechanical stimuli (Adrian, 1955). A similar mechanical sensitivity was reported also for the mammalian main olfactory epithelium, which was found to activate the MOB in response to odorants and also to the airstream pressure (Adrian, 1942). Responses to common odorants were later recorded in the VNO and its target areas in the brain (Tucker, 1963; Meredith and O'Connell, 1979), however, the idea emerged that the VNO could be sensitive to stimuli that included not only low molecular weight volatiles. According to data on the modulation of reproduction in mice and hamsters, it became clear that a direct contact of the snout with the chemical stimulus was mandatory for obtaining a modification of the hormonal milieu, in order to facilitate reproductive responses. For example, a peptide present in the urine of mice was proven to stimulate the anticipation of puberty in female mice via the stimulation of the VNO (Vandenbergh et al., 1975; Mucignat-Caretta et al., 1995), and the female pheromone in vaginal fluid of hamster was demonstrated to be a protein (Singer et al., 1986). This kind of stimuli could hardly be regarded as “volatile,” as confirmed by the necessity of direct contact of the snout with the pheromonal source, for the induction of pheromonal effects on the neuro-hormonal system. A major question regarded the type of stimulus that could access to the VNO, because it was not clear that high-molecular weight molecules could be transported inside the VNO lumen. At variance with the main olfactory epithelium, that receives the stimuli only via the airstream flowing through the turbinates, in the VNO an active vascular pumping mechanism allows large molecules dissolved in liquid to reach the organ (Meredith and O'Connell, 1979). By sucking up the liquid, large molecules or even particles are able to reach the VNO lumen (Wysocki et al., 1980; Mucignat-Caretta, 2010). Therefore, at least in some instances, the stimuli that activate the VNO are chemically different from those that activate the main olfactory system.

Lesion studies suggested a role for the accessory olfactory system in the modulation of reproductive physiology induced by pheromones: this is possible because the accessory olfactory system directly projects to the hypothalamus. However, a misunderstanding arose during the seventies, when several papers correctly pointed out that the VNO is connected with specific nuclei in the amygdala (Winans and Scalia, 1970; Scalia and Winans, 1975), and that information from the amygdala could modulate back the activity in the AOB, thus depicting an interactive circuit for the processing of inputs from the VNO (Raisman, 1972). Herein, the concept of a dual olfactory system emerged, with the main olfactory system devoted to the conscious perception of volatile odorants, due to its cortical projections, and the accessory olfactory system devoted to pheromones perception, due to its subcortical connections. Later on, this concept has been misinterpreted as an absolute specialization, and several studies assumed that information processing in the main and accessory olfactory system pertained to two different classes of stimuli, general odorants and pheromones, respectively. This is apparently not the case, since it is clear now that both systems are able to respond to either class of molecules, and that their central projections are heavily interconnected (Pro-Sistiaga et al., 2007), as summarized below.

#### Olfactory function during development

A possible distinction in the function of the main and accessory olfactory systems concerns their role in the first days of life. Immature newborns, like murine rodents, depend on tactile and olfactory stimuli for their survival. Several studies indicated that the main olfactory system is functional at birth, being involved in the pup's perception of the dam's pheromones (Teicher et al., 1980; Greer et al., 1982). In rabbit, the mammary pheromone is detected only by the main olfactory system (Charra et al., 2012), and in rat at least half of the main olfactory mucosa must be functional in order to drive the pup to the nipple (Kawagishi et al., 2009). Also cognitive functions related to olfaction are functional at birth. Olfactory learning in rodents is facilitated immediately after birth, in order to provide strong association among stimuli essential for survival (Miller and Spear, 2008). Processing of main olfactory signals, related to associative learning, is dependent upon the dense serotonergic innervation of the MOB mitral cells, that can be detected after birth (McLean et al., 1995). Olfactory learning is particularly facilitated during nursing, from feeding throughout the postprandial period: during this time, newborns easily learn associations between odors and other stimuli in order to acquire the characteristics of their familiar environment (Serra et al., 2009). However, the features of neonate olfactory learning are different from those of adults, suggesting a high degree of specialization in olfactory processing in the post partum period: for example, facilitation for learning of olfactory associations is accompanied by an increased difficulty in learning aversions during the first week, while the adult pattern of events emerges during the second postnatal week, in coincidence with the termination of the corticosterone hyporesponsive period (Moriceau et al., 2006). In the olfactory bulb, the refinement of electrical activity follows a three stage process: an immature period until day 11 of extrauterine life, a transition period until day 17 and a mature period thereafter (Sugai et al., 2005). A similar shift in activation pattern, revealed by c-Fos immunohistochemistry, has been detected in the rat piriform cortex (Illig, 2007). The modifications in olfactory processing in these two olfactory areas parallel neuro-hormonal and behavioral maturation. The responses to the same chemosignal, in fact, change during development of the receiver, as the hedonic value of male mice pheromones changes from infancy to puberty and adult age (Mucignat-Caretta et al., 1998).

The role of the accessory olfactory system during the first postnatal days is less clear than the role of the main olfactory system. The VNO is electrically active *in utero* (Mendoza and Szabo, 1988), but its functional role in the newborn is still obscure, albeit its structural characteristics suggest an advanced stage of functional maturation at birth (Coppola and O'Connell, 1989). Conceivably, in the first days of life, the main olfactory system has a major role in pup survival. However, recent evidence confirms that in mice the vomeronasal duct is open at birth and the sensory neurons do release neurotransmitter in the AOB already one day after birth; moreover, the precise connectivity of incoming axons is modulated by the exposure to pheromonal cues in the first postnatal days (Hovis et al., 2012). Contrary to previous data, this paper suggests a deeper involvement of the accessory olfactory system in chemosensory processing during the first postnatal days.

Likewise, the mother uses the olfactory system to discriminate its newborn. Apparently, the main role in the discrimination of the individual odor of each pup pertains to the main olfactory system (Keller and Lévy, 2012). Profound modifications in the synaptic circuits of the MOB take place at parturition, so that the mother learns her own pup odor, and shifts its behavior from aversion, as present in the virgin female, to maternal care (Lévy and Keller, 2009). However, maternal behavior onset benefits from both main and accessory olfactory information. In fact, the detection of dodecyl propionate, a pheromone produced by the rat pup preputial gland, is mediated by the VNO, thus triggering anogenital licking in the dam, a behavior necessary for the pup's survival (Brouette-Lahlou et al., 1999). Therefore, both the main and accessory olfactory systems are involved in mother-pup interactions.

## Functional overlapping of main and accessory olfactory systems in response to common volatiles and social odors or pheromones

Chemosensation mediates many aspects of social interactions in mammals not only during infancy but also afterwards, including sexual, territorial, and aggressive behaviors. The molecules that are involved in such interactions are not completely known, as well as their receptors. Different types of chemosensory receptors are present in the various receptors organs of the nose. They include the main olfactory epithelium, the VNO, the septal organ, and the Gruenenberg ganglion. The sensory neurons in these organs host various types of receptor molecules: olfactory receptors, V1R, the first class of vomeronasal receptors, V2Rs, the second class of vomeronasal receptors, trace-amine-associated receptors, formyl-peptide receptors, and guanylil-cyclase D receptors (for a review, see Fleischer et al., 2009).

The key distinction between main and accessory olfactory system led to the idea of a functional specialization of the two systems. However, from early studies it was clear that VNO stimulation did not coincide with pheromonal stimulation, since some pheromones could stimulate also the main olfactory epithelium (Meredith, 1998). The two systems instead differ in their projections to different amygdala nuclei, being the VNO linked to activation of medial amygdala (Samuelsen and Meredith, 2009), which in turn affects the luteinizing-hormone releasing hormone (LHRH) release from the hypothalamus. LHRH is released in female rodents after male chemostimulation of the accessory, not the main, olfactory system (Meredith, 1991), and involves long-term effects via the vomeronasal amygdala (Mucignat-Caretta et al., 2006). On the other hand, in both the main and accessory olfactory system vasopressin neurons are present to facilitate the social odor identification (Wacker et al., 2011): hence, the main olfactory system is mainly involved in learning and recognition of social odors for discrimination of individuals, pups, mates, or conspecifics (Sanchez-Andrade and Kendrick, 2009), while the accessory olfactory system may activate also the neurohormonal pathway. The sexual dimorphism of accessory olfactory system and the presence therein of steroid receptors support its involvement in reproductive/neuroendocrine modulation (Guillamon and Segovia, 1997). In turn, sociosexual interactions like mating may influence the rate of cell differentiation in the accessory, but not in the MOB (Corona et al., 2011).

Apparently, both the main and accessory olfactory system work together to allow successful social interactions: the first approach to airborne chemicals may be mediated by the main olfactory system, that in case of detection of interesting stimuli may trigger the active exploratory behavior, that is necessary for VNO pump activation (O'Connell and Meredith, 1984; Keverne, 2004).

### Various types of stimuli may activate the accessory olfactory system

The VNO handles different types of meaningful stimuli; however, stimulus processing appears more selective in the VNO, compared to the main olfactory epithelium (Luo and Katz, 2004). Chemical signals related to strain and individuals are encoded by populations of VNO neurons, while some cells respond to gender-specific cues (He et al., 2008). The VNO is also involved in the perception of alarm pheromones (Kiyokawa et al., 2007), a function it shares with the Gruenenberg ganglion (Brechbühl et al., 2008), and participates in the perception of predator chemosignals (Masini et al., 2010). The VNO may mediate also the behavioral reactions to non-pheromonal chemicals (Inagaki et al., 2010).

Vomeronasal neurons respond to known low-molecular weight urinary molecules (Del Punta et al., 2002) and volatile pheromones (Leinders-Zufall et al., 2000), mainly by activating V1 receptors (Boschat et al., 2002). However, vomeronasal neurons respond also to non-volatile chemosignals, like sulfated steroids (Nodari et al., 2008) and proteins, for example lipocalins like alpha-2U (Krieger et al., 1999) or peptides excreted by exocrine glands (Kimoto et al., 2005; Taha et al., 2009) or related to MHC (Leinders-Zufall et al., 2004). A class of VNO receptor cells express formyl-peptide receptors, which may respond to peptides from bacteria or related to the immune system (Liberles et al., 2009; Rivière et al., 2009). Some of the non-volatile chemosignals appear intrinsically attractive and may act as unconditioned stimuli for associated odorants (Martínez-Ricós et al., 2008).

Different populations of VNO receptors mediate responses to volatile pheromones and to proteins that are, respectively, perceived from cells bearing V1Rs, located in the apical part of the VNO mucosa, and from basal neurons, expressing V2Rs (Krieger et al., 1999). Complex mixtures involved in intraspecific communication, like urine, differentially activate various populations of receptors in the AOB (Dudley and Moss, 1999). Moreover, some cells in the apical layer of the VNO have been shown to express odorant receptors and project to the anterior AOB: they may sustain the responsivity of VNO/AOB to common odorants, in addition to cells expressing the vomeronasal receptors (Lévai et al., 2006).

Therefore, the VNO appears to be involved in the perception of different chemical stimuli, which may selectively activate various cells.

### Different types of stimuli induce various responses in the accessory olfactory system

VNO cells activity was firstly recorded in response to general odorants and also to volatile molecules that act as pheromones (Tucker, 1963; Meredith and O'Connell, 1979). Later on, the activity of the rodent VNO was investigated mainly by studying the responsiveness to putative pheromones, either volatiles or not (for a review, see Bigiani et al., 2005). The electrical activity induced by odorants was in general less studied, on the assumption that the main role of the VNO should be in the perception of pheromones. It is now clear that this assumption is an oversimplification of both the role of the VNO and of the signaling function of pheromones or social odors in intraspecific chemical communication.

In mice, the exposure to urinary odors leads to the activation of the VNO and the AOB, which respond differently from the main olfactory system. The responses to urinary stimuli appear to be mediated in VNO receptors by inositol-trisphosphate, while most olfactory receptors act via the cAMP cascade (Thompson et al., 2004). Different cells in rat and mouse VNO are selective for the urinary stimuli originated from each gender (Inamura et al., 1999; Holy et al., 2000). In fact, while main olfactory receptors are broadly tuned, the VNO neurons show high response selectivity: each pheromone activates a small subset of receptors that then converge on the AOB, in order to provide complex information already at the first step of central processing (Luo and Katz, 2004). At variance to the main olfactory system, in which cells expressing a single receptor converge to one glomerulus, VNO neurons expressing one specific receptor projects to different glomeruli, located nearby. In this way, each AOB glomerulus processes information coming from different receptors (Belluscio et al., 1999). In addition, single glomeruli and mitral cells receive inputs from different, but related V1R receptors, thus integrating information from various receptors (Wagner et al., 2006). The morphology of vomeronasal axons, which branch several times before reaching their glomerular targets, support divergent projections to different glomeruli (Larriva-Sahd, 2008).

The processing of VNO inputs in the AOB was initially compared to olfactory processing in the MOB, since both bulbs circuitry share some basic characteristics: the output cells are activated by peripheral receptors and in turn activate granule cells via dendrodendritic synapses, the mitral-to-granule synapse is glutamatergic, while the granule-to-mitral is GABA-ergic (Jia et al., 1999). However, the membrane responses of output cells in the AOB were found to differ from those in the MOB, so that the information processing in both bulbs appear more different than previously reported. Output cells in the AOB can be classified in three groups, according to their responses, and can be activated to amplify responses to long-lasting signals while depressing responses to short-lived stimuli (Zibman et al., 2011).

Both the main and accessory olfactory bulb may respond simultaneously to both odors and pheromones: volatile pheromones may strongly activate both bulbs. However, in the presence of complex mixtures of pheromones, like in the exposure to natural urine, the activation is more pronounced in the AOB than in the MOB, where the activation is limited to some regions, suggesting a different selectivity of the two systems (Xu et al., 2005).

Neurons in the AOB respond differentially to urinary pheromones (Guo et al., 1997), according to the gender of the donor and of the recipient: the AOB mitral and granule cells are more active in responding to opposite-sex urinary volatiles; these same stimuli concurrently activate discrete clusters of MOB glomeruli, in both males and females (Martel and Baum, 2007; Baum, 2012). However, the activation of the accessory olfactory system is not mandatory to discriminate the urine derived from males or females with different hormonal status, and does not prevent the expression of male sexual behavior. The AOB appears indeed important for coding the incentive value of opposite-gender urinary volatiles (Jakupovic et al., 2008). Apparently, the processing of pheromonal inputs, mediated via the accessory olfactory system, acts in females to suppress the male-typical courtship behavior, through gender-specific connections in the amygdala and hypothalamus (Baum, 2009).

Both the main and accessory olfactory system processing of social chemosignals are influenced by the hormonal status, including steroid and non-steroid hormones, which modulate the responses to chemical cues. This modulation involves behavioral modifications, by enhancing investigation, or neural mechanisms that enhance the responsivity of both the main and of the accessory olfactory bulb to the specific chemosignals, to provide concurrent stimulation that enhances the probability of mating in the most favorable conditions (Moffatt, 2003). Therefore, the main and accessory olfactory systems simultaneously process complex chemosensory stimuli according to the neurohormonal status of the receiver.

## Convergence of main and accessory olfactory inputs within the brain

The abovementioned studies show that both the main and accessory olfactory systems process similar stimuli, but their involvement appears slightly different in inducing various effects. Therefore, it is important to understand how, when and where the two systems integrate their respective information.

The central olfactory projection areas were initially investigated by functional studies that revealed several brain areas connected to the processing of olfactory inputs (Powell et al., 1965).

Anatomical tracing studies revealed that the main olfactory epithelium and the VNO projected to the main and accessory olfactory bulb, respectively (Barber and Raisman, 1974).

The processing of odorant information flows then from the MOB, where the single mitral cells are activated by few odorants, to the anterior olfactory nucleus, where neurons show a broader responsiveness, and where responses to mixtures often exceed the responses to the single components (Lei et al., 2006). In the MOB, the axons of cells expressing the same receptor do converge to couples of glomeruli, defining a stereotyped map. In the piriform cortex, however, no spatial map can be recognized for mapping odorant identity (Choi et al., 2011). This is due to a different wiring of homotypic mitral/tufted cells (Ghosh et al., 2011), which project diffusely from MOB to the piriform cortex (Miyamichi et al., 2011; Sosulski et al., 2011).

The segregation of vomeronasal inputs starts at the peripheral level: V1R receptors are hosted in the more superficial layer of the vomeronasal epithelium, and V2R neurons are located deeper; the afferents remain segregated by projecting to the anterior and posterior AOB, respectively (Bigiani et al., 2005; Tirindelli et al., 2009). A certain degree of separation remains also in the downstream projection areas, since the anterior AOB is connected to the bed nucleus of the stria terminalis, and the posterior AOB projects to the dorsal anterior amygdala, and remains in part segregated also in the hypothalamus (Mohedano-Moriano et al., 2008). The amygdala is involved in the modulation of fear and anxiety behavior as well as intraspecific reproductive and aggressive behavior (Martínez-García et al., 2008); it receives inputs directly from the AOB, and is involved in the perception of sexual pheromones, giving them an innate attractivity, independently from the activation of the reward system in the ventral tegmental area. In turn, the amygdala project to the striatal olfactory tubercle and Islands of Calleja, which are presumably involved in pheromonal communication (Lanuza et al., 2008). The cortex is a site of convergence of segregated VNO pathways, since no segregation has been detected in accessory olfactory cortical areas, including the bed nucleus of the accessory olfactory tract, the medial amygdaloid nucleus, the posteromedial cortical amygdaloid nucleus, and the bed nucleus of the stria terminalis (Von Campenhausen and Mori, 2000).

The interaction between the main and the accessory olfactory systems may initiate already at the first stages of central processing. The AOB receives all chemosensory inputs from the VNO. However, the principal cells of the AOB and the bulbar interstitial neurons, hosted in the most rostral portion of the AOB, allocate efferent fibers originating from the MOB, as demostrated by terminal degeneration in the AOB after MOB lesions, being thus the first documented site of convergence of main and accessory olfactory inputs (Larriva-Sahd, 2008).

The crosstalk between the main and the accessory olfactory system proceeds also in the subsequent stations: single neurons in the amygdala may receive inputs from both systems (Licht and Meredith, 1987), and the medial amygdala, which is a target for AOB projection neurons, also receives inputs from the MOB in its more superficial laminae (Kang et al., 2009).

**Figure 3:**
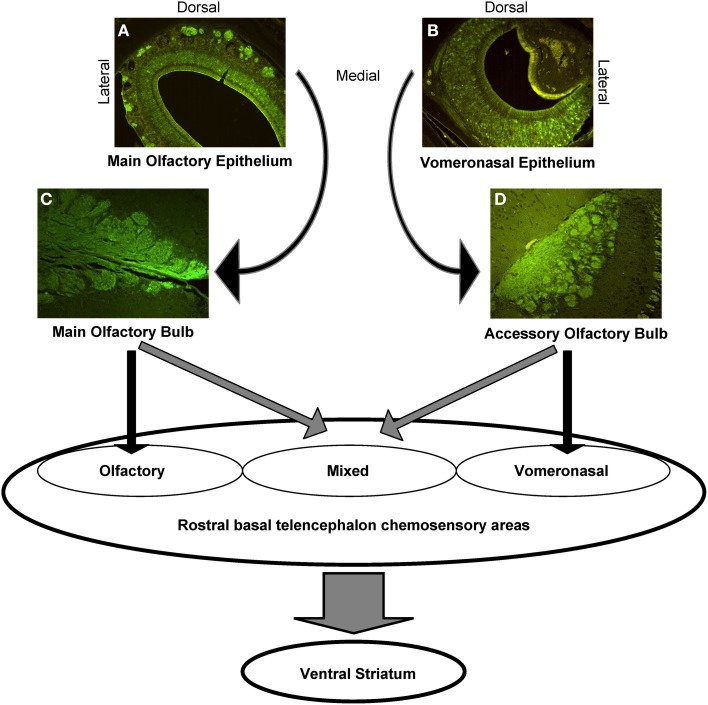
**Schematic representation of the central connections of the main and accessory olfactory systems.** The photomicrographs show the anti-Olfactory Marker Protein immunoreactivity, that highlights the main olfactory neurons and nerves **(A)**, and the glomeruli in the MOE **(C)**; the VNO sensory neurons **(B)**, and the AOB glomeruli **(D)**.

Apparently, the interconnection of the main and accessory olfactory system delineates also areas in which the systems do overlap, including the anterior medial amygdala and the stria terminalis. The two systems converge also in classical olfactory areas, including the nucleus of the lateral olfactory tract, the anterior cortical amygdaloid nucleus, and the transition between amygdala and cortex, but also in previously considered exclusive vomeronasal-receiving areas, including the ventral anterior amygdala, the bed nucleus of the accessory olfactory tract, and the anteroventral medial amygdaloid nucleus. In other areas, however, the main and accessory olfactory inputs remain segregated, for example in the posteromedial and posterolateral cortical amygdala: consequently, a functional distinction between olfactory, vomeronasal and mixed chemosensory areas has been proposed (Pro-Sistiaga et al., 2007, see Figure 3).

The centrifugal pathways, which send back information to the various areas, play a great role in the processing of chemosensory inputs (Mohedano-Moriano et al., 2012). Ideally, the schema of vomeronasal projections follows a simple three-step process from VNO to AOB and hypothalamus, supporting the modulatory role for reproductive processes. The AOB receives inputs from neurons hosting estrogen receptors therefore, a modulation of incoming stimuli is present already at the level of the AOB, according to the hormonal status. Actually, the bed nucleus of the stria terminalis sends GABAergic axons to the AOB mitral cells, while the vomeronasal amygdala sends glutamatergic inputs to the AOB granule cells (Fan and Luo, 2009). Also the olfactory-recipient medial amygdala has been proposed to send back information to the AOB (Martel and Baum, 2009). Moreover, the entorhinal cortex receives afferents from the MOB and in turn sends to the hippocampus that projects back from the CA1 field and the ventral subiculum, to the AOB, suggesting a possible involvement of memory-related areas in AOB processing (de la Rosa-Prieto et al., 2009).

Thus, the primary projections to the main and accessory olfactory bulb remain separated, while the secondary projections to the rostral basal telencephalon and the tertiary projections to the ventral striatum widely overlap (Martinez-Marcos, 2009).

## Conclusions

Social interactions in numerous species of mammals are mediated by a variety of different chemical stimuli, which are concurrently perceived by the main and accessory olfactory system. Both systems may process similar chemosignals, and participate in managing the reproductive behavior, from mate identification to pup care (Keller et al., 2009). Both systems are possibly involved in the detection of biologically relevant odors, by processing them in parallel and contributing to the different neurohormonal responses at various degrees (Restrepo et al., 2004; Spehr et al., 2006). A possible complementary function may be foreseen for the two systems, with the main olfactory system involved in detection of substances at a distance, and the vomeronasal system having a more specific role in active exploration of relevant stimuli (Martínez-García et al., 2009).

In every instance, the cross-talk between the main and the accessory olfactory systems from the early stages of central processing, as demonstrated by the anatomical connections, supports the idea that both systems participate in the construction of an unitary chemosensory perception.

### Conflict of interest statement

The authors declare that the research was conducted in the absence of any commercial or financial relationships that could be construed as a potential conflict of interest.
